# Procedures and potential pitfalls for constructing a bee-infecting RNA virus clone

**DOI:** 10.3389/finsc.2022.908702

**Published:** 2022-09-26

**Authors:** Wei-Fone Huang, Runlin Li, Lang Jin, Shaokang Huang

**Affiliations:** College of Animal Science (College of Bee Science), Fujian Agriculture and Forestry University, Fuzhou, China

**Keywords:** reversed genetics, SBV, DWV, honey bees, picornalike virus

## Abstract

Viruses are factors that can fluctuate insect populations, including honey bees. Most honey bee infecting viruses are single positive-stranded RNA viruses that may not specifically infect honey bees and can be hazardous to other pollinator insects. In addition, these viruses could synergize with other stressors to worsen the honey bee population decline. To identify the underlying detailed mechanisms, reversed genetic studies with infectious cDNA clones of the viruses are necessary. Moreover, an infectious cDNA clone can be applied to studies as an ideal virus isolate that consists of a single virus species with a uniform genotype. However, only a few infectious cDNA clones have been reported in honey bee studies since the first infectious cDNA clone was published four decades ago. This article discusses steps, rationales, and potential issues in bee-infecting RNA virus cloning. In addition, failed experiences of cloning a Deformed wing virus isolate that was phylogenetically identical to Kakugo virus were addressed. We hope the information provided in this article can facilitate further developments of reverse-genetic studies of bee-infecting viruses to clarify the roles of virus diseases in the current pollinator declines.

## Introduction

Honey bees are critical pollinators in agricultural productions and natural environments, but the populations were in decline trends in various regions (1). Viral diseases are natural modulators for insect populations and stress factors link to pollinator population declines (1, 2). Although most honey bee infecting viruses were firstly identified in honey bees, these viruses may not originate from honey bees. Moreover, honey bee infecting viruses can spill over to other wild pollinators and insects (3, 4). Most well-studied bee-infecting viruses are single positive-strand RNA viruses with a simple genome composition of 8-11 kb sizes. These RNA viruses belong to the Picornavales order and are similar to picornaviruses found in other animals and cause diseases that resulted in economic losses and death. Compared to picornaviruses in humans (e.g., poliovirus and enterovirus) and other animals (e.g., Foot-and-mouth disease virus), bee-infecting viruses are much lacking in reversed genetic studies. Reversed genetics reveals detailed viral gene functions, virus competition (5, 6), transmission (6–9), virus/host interactions using sequence manipulated virus infectious cDNA clones.

Only a few infectious cDNA clones of bee-infecting RNA viruses were reported after more than 40 years of the first infectious picornavirus clone (10). Black queen cell virus (11), Deformed wing virus [(12), Austrian DWV-A; (6), American DWV-A; (9), American DWV-B], Chronic bee paralysis virus (13), and Sacbrood virus (14) were cloned and demonstrated to be infectious (details and additional clones were listed in **Table 1**). In our previous study (14), we accomplished the cloning of SBV, but the same protocol was somehow difficult for cloning a local Deformed wing virus isolate that phylogenetically grouped with Kakugo virus (16). We have investigated the factors that may affect cloning, and we would like to further discuss the cloning processes and the potential pitfalls in steps of cloning bee-infecting RNA viruses in this article. We keenly hope this article will be useful to researchers who are interested in applying virus infectious cDNA clones within honey bee studies.

**Table 1:** Published cDNA infectious clones of honey bee picorna-like viruses.

	Family	Subtype (location)	Infectiousness	References
BQCV	Dicistroviridae	BQCV (South Africa)	Infectious	(11)
CBPV	undecided	CBPV (Austria, Vienna)	Infectious	(13)
		CBPV (China, Hubei)	Likely infectious*	(15)
DWV	Iflaviridae	DWV-A (Austria)	infectious	(12)
		DWV-A (USA, Maryland)	Infectious	(6)
		DWV-A and DWV-B (UK)	Infectious	(8)
		DWV-B (USA, California)	Infectious	(9)
IAPV	Dicistroviridae	IAPV (China, Beijing)	Likely infectious*	(15)
SBV	Iflaviridae	SBV (China, Fujian)	infectious	(14)

*The infectiousness was mainly demonstrated by qRT-PCR, lacking distinguishable label and wild-type virus-free controls. Overall infectiousness of the clones was less vigorous than that in other studies.

## Obtaining cDNA templates

Honey bee infecting RNA viruses have small RNA genomes that are not technologically challenging to obtain the full-length cDNA template. Commercially available reverse transcriptase enzymes can synthesize cDNA in 10 kb sizes, which is more than enough to synthesize the full genome; however, the virus isolations and the viral genome termini sequences are potentially problematic.

Virus isolation and enrichment is the common first step for cloning, but this step can be difficult for bee-infecting viruses. Similar viruses of mammals can be isolated and propagated using culture methods; in brief, this method transfers a tiny infected tissue into the suitable and virus-free cell or animal hosts and then is followed by subcloning processes to select infected cells/animals that can be passaged for virus propagation. Virus isolation using cell cultures is much less costly and quite advantageous for covert infections that may have limited virus quantities and multiple virus co-infections from the host (17). Although covert and multiple co-infections are commonly found in honey bees, immortalized cell lines that are easy to be applied have not yet been widely available for the viruses, and virus-free honey bees were seldomly found in studies. AmE-711, the published honey bee cell line, has DWV contaminations (18). Although infecting primary cultures of bee tissues are workable host cells for bee viruses (19–23), lacking virus-free honey bees can curb the decision of developing a primary culture. Moreover, the instabilities of primary cultures can be frustrating and difficult to repeat. Common lepidopteran insect cell lines may have difficulties serving as alternative hosts for selecting and proliferating bee-infecting virus isolates or infectious cDNA clones. *Spodoptera frugiperda* cell lines, one of the widely available insect cell lines, may not host the viruses (24, 25). We have transfected the full-length SBV RNA from our previous work (14) into Sf9 cells and identified the produced virions using RT-qPCR with RNase treatment in preliminary trials, but we found the produced virions were low in quantities and not able to be passaged in Sf9 cells, similar to what has been published by (25). The produced SBV virions seem not to be able to enter Sf9 cells naturally. The current easy solution for honey bee virus isolation was isolating the virus from a small number of bees, the fewer the better, to reduce the risk of contamination of multiple virus species.

Bypassing the virus purification step may be another feasible solution for some bee-infecting viruses. It was a common strategy to simplify cloning processes if highly infected samples are available (26). For DWV and SBV that can create obvious overt infections, it is possible to skip the virus isolation and enrichment step and use the cDNA from a single honey bee to clone the full-length viral genome. Viral genome fragments can be PCR amplified from the cDNA of an infected larva or worker bee and assembled into a full-length genome. We have used the cDNA sample from a single diseased larva to amplify SBV genome fragments, approximately 3 kb in size, that were later assembled into a full SBV genome cDNA template (14). Another attempt to amplify a nearly full-length DWV genome using a cDNA sample isolated from a worker with atrophied wings was a success (16) (published in Chinese), which is similar to that in (6) used a DWV isolate from 50 pupae. In brief, we selected a cDNA sample that generated a low DWV Ct value, the Ct value was approximately 15 which was lower than the Beta-actin in qPCR results. A primer set that targets the entire DWV ORF and partial UTRs, approximately 10 kb in size, was designed to amplify the 10 kb fragment with a modified touchdown PCR program, which started by using the annealing temperature two degrees Celsius higher than the estimated Tm of the primers and ended at two degrees Celsius lower than the annealing temperature, one degree Celsius decreased every five cycles, and then followed by 20 cycles of the lowest annealing temperature. This nearly full-length DWV genome PCR product was used as an enriched viral cDNA template, which has greatly facilitated the following cloning works that required many steps of trial and error. This result also suggested that skipping the virus isolation and enrichment step is workable in bee-infecting virus studies if heavily infected bees are available.

Another limitation for obtaining a full-length cDNA template for desired bee-infecting RNA virus could be the termini sequences. Viral genome termini were untranslated regions (UTR). Since most picornavirus and picorna-like virus genome structures are similar to mRNA, rapid amplification of cDNA ends (RACE) was used to obtain the terminal sequences. 3’ RACE was relatively easy compared to 5’ RACE (27). In addition, the 3’ UTR termini can be identified by the poly (A) region. If there is any concern about the completeness of 5’ UTR, an additional 5’ RACE may be needed, similar to what has been done for DWV (12, 28). Alternatively, adapting the 5’ UTR sequence of the type genome found in databases or closely related virus isolates is a viable option if the 5’ UTR is not the research target. For example, Gusachenko etal. (8) used identical termini sequences for all cloned isolates, and we used the UTRs of the SBV genome previously isolated from the same place (14).

Termini UTR regions form secondary structures (29), e.g. internal ribosome entry site (IRES) of 5’ UTR for initiating translation, which could create difficulties for PCRs that used termini sequences as the primers. In addition, we noted DWV and SBV have A/T rich 5’ end terminal sequences that are difficult to be used as a primer. Our solution was to select the primer annealing sites close to the termini and still have good properties for primer designs, and the termini sequences can be synthesized through oligo or gene synthesis services. Synthesized termini can be assembled into the viral cDNA template later. Gusanchenko et al. used a vector that has cloned termini sequences (8). In our previous work (14), we used anchored PCR (additional sequences were added at the 5’ end of the primers) to add the termini sequence before the assembly of the full viral cDNA template. It should be practical for adding sequences in sizes of 40-60 bp using anchored PCR, and primer (single oligo DNA) synthesis services can be commonly found and outsourced internationally.

The assembly of the PCR amplified viral cDNA is usually accomplished by restriction (30) or Gibson cloning methods (12, 14, 31, 32). The restriction cloning method has been widely applied in plasmid constructions that used restriction-enzyme sites to create complementary sticky ends on the targeted DNA fragments for assembly using DNA ligase. This method is robust but limited by the existence of restriction-enzyme sites on the targeted DNA fragments if altering sequences or adding additional sequences having a restriction-enzyme site is not desirable. Gibson cloning method (33) has been widely applied in recent virus cloning because of its easy applications with no need for adding or altering sequences. The method utilizes a mixture of three enzymes, exonuclease, ligase, and DNA polymerase, working together at the same temperature. The DNA fragments need to have overlapped sequences (approximately 15-60 bp) on the termini that will be digested by the exonuclease into complementary sticky ends, the gaps will be filled by the DNA polymerase, and then the ligase will join the DNA fragments. Various commercial kits are available with detailed information and suggestions.

Large-scale production of the assembled virus genome template can be trickier than regular DNA fragments in similar sizes. A large amount of the templates, in micrograms, would facilitate *in vitro* transcription of the viral RNA for transfection. The most common method is cloning the viral cDNA template into a vector to form a circular plasmid that can replicate within *Escherichia coli*. This method is often used because of the easiness and the cost for large-scale productions, but the plasmid containing viral sequences might be unstable in *E. coli* (32, 34). The instability is not yet fully understood, and the common processes to avoid the instability include reducing *E. coli* metabolic burdens, limiting plasmid copy numbers, increasing selection pressures, and selecting *E. coli* strains that could tolerate the viral genome fragment (34). We have tested several commercially available *E. coli* strains that potentially have higher tolerances for toxic and viral genes, including HB101, Stbl, Stbl2, Stbl3, and SURE (16). We found the plasmid that contains the cloned SBV genome (14) can be stable using pBR322-derived vectors (approximately 5-10 copies within *E. coli*) in the tested strains, and other studies used TOPO (6) and pACYC177 (15) vectors. Nevertheless, our full-length DWV genome (Fujian isolate) cloned using the same pBR322 vector has a higher chance to be mutated in all tested strains (16). We have used a low culture temperature (30°C) for the bacteria culture according to the suggestions from the vendors of the *E. coli* competent cells, but the instability still randomly occurred, including in scaling up the culture. The conditions of large-scale production may need to be carefully controlled. A preliminary trial to find the growth curves in the culture mediums and the temperatures may be needed, and temperatures that are lower than 30°C may be used to reduce the instability of some viruses. We have tested LB and SOC medium for *E. coli* culture, and we found SOC has a slight but insignificant enhancement. We also noted the mutations during the large-scale production are randomly deleting a huge chunk of the viral genome according to the sequencing results (16). Such mutations can be revealed in gel electrophoresis; therefore, we always conduct gel electrophoresis of the plasmids.

Instabilities of the plasmid contained DWV genome cDNA within *E. coli* have not been noted in previous studies cloning DWV subtype A (6, 31) and subtype B (7, 9). DWV was suggested to be a quasi-species that included two formally recognized subtypes, DWV-A (the original DWV) and DWV-B (originally named *Varroa destructor* virus-1, VDV-1), and a recently identified DWV-C (35). Our cloned DWV genome was phylogenetically grouped with Kakugo virus with many East-Asia DWV isolates (16), similar to that in (6). Kakugo virus shares more than 97% similarity to DWV-A (36, 37), but Kakugo virus seemed to cause aggressive behaviors that have not been identified in DWV-A infections (38). Interestingly, the Kakugo virus genome was revealed earlier than that of DWV-A, but no infectious cDNA clone of Kakugo virus has been used in studies. The instability issue may be overcome with trial and error in months, testing vectors, *E. coli* strains, and culture conditions (16), but it may have curbed the studies of Kakugo virus and other DWV strains phylogenetic closely related to Kakugo virus in East-Asia. Genetic instability of honey bee RNA viruses is a factor that should be considered in future study designs, including cloning and constructing virus isolate libraries through cloning. Some viral cDNA instability problems may be conquered by adapting the methods recently used in constructing a SARS-CoV-2 clone (30). They used a single-copy plasmid, i.e. CopyControl pCC1BAC Vector, to reduce the chance of mutation during replicating the plasmids within *E. coli* and separate the viral genome into two fragments by a restriction enzyme site that can be assembled by T4 ligase before *in vitro* transcription.

## Full-length viral RNA preparations

Bacteriophage RNA polymerase (T7 or SP6) can transcript the full-length viral RNA. We found that T7 RNA polymerase kits with the specifications stating the capabilities of synthesizing long RNA fragments are convenient and widely available from vendors. The transcription usually needs approximately 1,000 ng of linearized viral cDNA templates and generates more than 30 μg of viral RNA after simple purification using alcohol precipitation. We noted the RNA quantities measured using nanodrop were sometimes higher than that estimated in gel electrophoresis using a fluorescent dye, similar to the difference between qubit and nanodrop reported (39). Qubit or similar bioanalyzers that provide accurate RNA quantifications can be helpful here.

In-cell transcription of the viral RNA has not been found in bee-infecting virus studies, but it has been used in picornavirus clones. In-cell transcription has facilitated experiments using *in vitro* cultured cells and animal trials (32, 40) because fewer steps were needed and DNA is easier to process compared to RNA. CMV promoters or other promoters originating from insect DNA virus sequences (IE1 and IE2) may be useful to construct in-cell transcription of bee-infecting virus clones, but further tests of these promoters in honey bees will be needed. Liposomes or other transfecting reagents will be needed for transfecting DNA into honey bee cells. Although in-cell transcription may simplify the workflow, the transfection protocol and the formula to mix liposomes may need to be optimized with additional efforts.

## Transfection of the viral RNA

Naked viral RNA can infect honey bees without transfecting reagents (6, 8, 12, 14, 31). RNA can be ingested to trigger RNAi responses in honey bees (41). This remarkable RNA intake phenomenon might relate to antiviral responses in bees. Delivering the viral RNA into bees still matters because RNA degradation may happen during the delivery. We think oral feeding of naked full-length viral RNA may not be as easy as short ones that are used in RNAi. In the cloning of SBV (14), oral feeding and injecting methods to transfect transcribed viral RNA on bee larvae have been tested. Although oral feeding seems to be the easy choice and was reported in a previous study (12), we found some limitations. Regular artificial larva diet consists of yeast extract, sugars, and royal jellies that harvest from honey bee hives. Although a royal jelly major protein stabilized RNA (42), the microbes and the digestions within the gut may fragment the long RNA fragments. Royal jellies may be contaminated by viruses and RNases from the labor-intensive harvest processes. Furthermore, bee larvae have a small time window, 3- to 5-day-old larvae, for transfection although it would be varied according to the virus species and study designs. Larvae younger than 3-day-old are quite small to graft and feed. Larvae older than 5-day-old may enter the prepupae stage within the desired incubation time. Pupation may add additional variables to viral infection intensities and propagation efficiencies in insects. Viruses that cause only overt infection in larvae may not propagate well in pupae, e.g., we noted that SBV clones might not be as infective in pupae compared to larvae in preliminary trials. We had no success results in a few attempts of oral feeding of viral RNA with a regular artificial bee diet. We also found it difficult to add a starving time for young larvae that may encourage them to consume the diet within a short time. In addition, we were afraid that starving may affect the developments. The idea of injecting larvae was a compromised solution because of causing a needle wound on the fragile larval cuticle. We did note a high death rate after the injection, but all survived larvae have detectable cloned virus infection (14).

In summary, full-length viral genome RNA can be transfected in the naked form, and the pathway for delivery of the viral RNA into cells was the major potential issue that need to be addressed in the study design. For viruses causing only overt infections in larvae, transfecting the RNA into larvae may be through either feeding or injecting; feeding has higher risks for RNA degradation and unevenly dosing, and injecting creates an additional wound that may elicit immune responses and increase mortality. For viruses causing overt infection in pupae, injection of pupae would be the choice. To avoid virus contaminations from field-collected larvae and lead misinterpretations of the results, a proper control group that injected inviable viral RNA fragments would be needed.

## Validations of the infectivity

Infectivity of the virus clones needs to be evaluated using a bioassay using bees. Virus-free or relatively virus-free bees should be the first thing to be considered in bioassays. Most of the testing bees used in published studies were still harvested from colonies kept in fields with uncontrolled exposures to the environments. In addition, the viral infections in honey bees followed the first-come-first-serve order that the viral strain firstly infects the host can dominate the competition (5), which was also noted in other insect pathogens (43). Therefore, wild-type virus contaminated bees should be prevented in a bioassay. We noted the wild-type of the cloned virus can affect the bioassay results in our previous SBV study (14). The dynamics of the diseases within a colony could be altered rapidly according to environmental changes and manipulations. Virus screenings right before harvesting bees to exclude the wild-type infections from the colonies would be necessary to prevent unpredictable interferences in either the control or the experiment groups. Before harvesting bees for trials, the colonies were first visually screened to exclude the colonies with *Varroa* infestation and other signs of virus infections. Then the colonies were sampled for qPCR diagnosis to reveal covert infections, more than 50 individuals were needed from each colony (44). We usually pooled 5-10 bees to save cost and time in RT-qPCR screenings. Prevalence of the targeted virus species may also be a factor in the screening. We can find bee larvae free of SBV infection or having really low infection rates and intensities to conduct needed trials, but DWV-free bees seemed to be much more difficult to find, even in *Apis cerana* colonies without *Varroa* mites.

Validations of cloned virus infection and replication in bioassays are similar to those used in regular virus infection studies. The bioassay study design needs to be rigorous with competent control sets, including a blank control group for revealing any covert infections that could turn into overt infection within the trial and a negative control group that received a defected virus clone. The host stage (ages of larvae, pupae, and adults) and sampling tissues should be properly selected for the cloned virus to obtain consistent results in biological repeats of the bioassay study. In addition, non-lethal sampling methods (45) for adult bees may provide the possibility to evaluate the infection of the same bee over incubation time if the virus exists in the hemolymph. Sampling hemolymph through clipping legs is commonly done in insect studies. Among the methods exam positive-strand RNA virus infectivity, qRT-PCR and negative-strand detection were the most common methods. Increasing trends of virus quantifications using qRT-PCR in bioassays can be evidence for clone infections. Since negative strands only exist in an intermediate stage of replications to serve as templates for viral RNA genome syntheses within host cells, the negative strands are solid evidence for the infections in studies; nonetheless, the negative strands exist in a relatively small amount compared to the positive viral RNA genome (46, 47). Specific primers with tags may be needed in reverse transcription to facilitate the negative strand detections (48) . High viral infection intensities and little RNA degradations of the samples would increase the possibility of detecting the negative strands. However, the negative strand detections can be misleading in some particular cases; for example, *V. destructor* samples may contain intact negative strands within ingested honey bee cells (49).

Adding distinguishable labels to the clone should be considered to facilitate infectivity validations. Synonymous mutations within the ORF (12) were used as labels to distinguish clones from wild-type viruses in the bioassays. Such labels can be added by alterations of the sequences during PCR amplification and assembly, and these labels need either a PCR or sequencing to identify. Epitope tags are often fused with *in vitro* produced proteins for labeling but not many found for labeling cloned picornavirus (50). Epitope tags are small peptides, 6-15 amino acids, that can be fused with targeted genes and then recognized by commercially available antibodies. No infectious clone of honey bee infecting viruses was yet able to express an epitope tag fused gene. Identification of the regions within the viral genes that can tolerate the fusion of epitope tags may be necessary. Fluorescent proteins are common labels that can be either fused or independently expressed in virus clones. Several different methods have been used to add EGFP to the clones. A Seneca Valley virus clone fused EGFP sequence to 2A protein (51). EGFP expression has also been added to bee-infecting virus clones, DWV and SBV clones. The DWV clone used repetitive proteinase recognition sites to add an independent EGFP expression in the genome ORF (31), and the SBV clone used IGR-IRES to express an EGFP in the 3’ UTR (14). These two clones both have capabilities to express foreign genes, and fluorescence proteins, mCherry (5) and nanoluciferase (52) were expressed by the DWV clones. The reason that we selected 3’UTR was because of several failed attempts of fusing epitope tags to SBV genes. We were not able to identify the reasons for the failed clones, possibly caused by epitope tag properties, interferences of the gene, and viral polyprotein cleavages. At the same time, we have added a restriction enzyme sequence at the site between 3’UTR and poly(A) without affecting the clone viability. Therefore, we decided to try the idea of adding IGR-IRES from a dicistrovirus in the 3’UTR to express an additional gene, which imitates the genome structure of dicistroviruses. More trials have identified that SBV 3’UTR is not critical for replications, and a gene of approximately 1.5 kb size was added to the same site without affecting the clone viability (unpublished data). The plasticity of the SBV 3’UTR is interesting, but further studies would be needed to identify if similar plasticity exists in other viruses.

## Conclusion

Current studies of bee-infecting RNA virus clones have paved the way for further studies of reversed genetics and applications. The virus cloning processes are relatively straightforward. With incoming honey bee cell lines (53), efforts in finding alternative host cells, and clarifications of viral polyprotein cleavages, more applications of virus clones in honey bee research are expected, including anti-virus responses, virus/host interaction, and ecology. Such studies may elucidate the role of virus diseases in population declines of domesticated honey bees, wild bumble bees, and other pollinators that are prone to bee-infecting virus infections. We hope the design rationales and the experiences shared within this perspective can alleviate some intimidation for other researchers who have little experience in bee virus cloning studies.

## Author contributions

WF-H drafted the manuscript. RL, LJ, and SH assisted in the data collection. All authors contributed to the article and approved the submitted version.

## Funding

This article was supported by in-school grants (1329000106 and 722022013) from College of Animal Science (College of Bee Science), Fujian Agriculture and Forestry University.

## Conflict of interest

The authors declare that the research was conducted in the absence of any commercial or financial relationships that could be construed as a potential conflict of interest.

## Publisher’s note

All claims expressed in this article are solely those of the authors and do not necessarily represent those of their affiliated organizations, or those of the publisher, the editors and the reviewers. Any product that may be evaluated in this article, or claim that may be made by its manufacturer, is not guaranteed or endorsed by the publisher.
